# Changes in Circulating Monocyte Subsets (CD16 Expression) and Neutrophil-to-Lymphocyte Ratio Observed in Patients Undergoing Cardiac Surgery

**DOI:** 10.3389/fcvm.2017.00012

**Published:** 2017-03-15

**Authors:** Kareem Gawdat, Stephanie Legere, Chloe Wong, Tanya Myers, Jean Sylvia Marshall, Ansar Hassan, Keith R. Brunt, Petra C. Kienesberger, Thomas Pulinilkunnil, Jean-Francois Legare

**Affiliations:** ^1^Department of Pathology, Dalhousie University, Halifax, NS, Canada; ^2^Department of Microbiology and Immunology, Dalhousie University, Halifax, NS, Canada; ^3^New Brunswick Heart Centre, Saint John, NB, Canada; ^4^Dalhousie Medicine New Brunswick, Saint John, NB, Canada

**Keywords:** REACH project, biomarkers of inflammation, CD16 expression, intermediate monocytes, phenotypic shift, NLR, cardiac surgery, non-classical monocytes

## Abstract

**Background:**

The characteristics of circulating inflammatory cells (leukocytes) in patients undergoing heart surgery remains poorly understood. Recently, neutrophil-to-lymphocyte ratio (NLR) and specific monocyte subsets (based on CD14/CD16 expression) have been suggested as markers of inflammation and predictors of outcomes. The present study aims to characterize the influence cardiac surgery with cardiopulmonary bypass has on specific circulating leukocytes.

**Methods:**

All enrolled patients had blood samples taken pre- (0 days), early post- (5 days), and late post- (90 days) surgery. Complete blood counts were performed and whole leukocyte isolations were obtained from blood samples and analyzed with flow cytometry. Fluorophore-linked antibodies (CD45, CD11b, CD14, and CD16) were added to the blood cell isolations and later assessed by flow cytometry.

**Results:**

Seventeen patients were enrolled and samples obtained at 0, 5, and 90 days. We demonstrated a significant increase in NLR (2.2-fold; *p* = 0.0028) and CD16 mean fluorescence index (MFI-measure fluorescence intensity shift of CD16 in a gated cell population) early at day 5 (2.0-fold; *p* = 0.0051). Both NLR and CD16 MFI levels generally returned to normal by day 90. There was a significant positive correlation between NLR and CD16 MFI (*r*^2^ = 0.29; *p* = 0.0064). Adverse cardiovascular event (AE) was defined as prolonged length of hospitalization or readmission to hospital for cardiac reasons after discharge was seen in 59% of patients (no deaths occurred). In an unadjusted analysis of AE, we identified NLR as a likely predictor of AE, which meant that patients developing AE had a significantly higher baseline NLR (*p* = 0.0065), something that was not observed with CD16 MFI (*p* = 0.2541).

**Conclusion:**

Cardiac surgery is associated with a significant increase in NLR and CD16 MFI (non-classical monocytes) early after surgery corresponding to the early inflammatory phase after surgery. Furthermore, we have, for the first time, identified a significant correlation between NLR and CD16 MFI. While the mechanism for this relationship remains unclear, our findings support the use of a simple test of NLR as a biomarker of inflammation for predicting outcomes in cardiac surgery patients.

## Introduction

The role of inflammation and its effector cells (circulating leukocytes) in patients undergoing heart surgery remains poorly understood. Traditionally, it has been difficult to differentiate between the normal beneficial inflammatory response needed for healing and components of the inflammatory response that can be linked to disease progression. There is increasing evidence that changes in circulating leukocytes associated with disease hold important prognostic information that could be used clinically (1–5). In particular, cardiopulmonary bypass (CPB), which is used extensively in patients undergoing heart surgery, has often been suggested as an important inflammatory stimulus responsible for significant changes in leukocytes that remain poorly defined (6–8).

Neutrophil-to-lymphocyte ratio (NLR) obtained from patients prior to surgery has been reported to be an independent predictor for the development of adverse events in patients undergoing cardiac surgery (1, 2, 5). This is also true for patients with acute coronary events and not specific to heart surgery, suggesting that NLR may represent a measure of the burden of inflammation in patients (9). The mechanism by which NLR is linked to patient outcome remains unclear. However, some investigators have suggested that major stress, such as surgery, results in significant changes in neuroendocrine factors such as cortisol, which could be responsible for the associated lymphopenia and affect NLR (10).

Similarly, the relative proportion of specific monocyte subsets has been linked to adverse outcomes in patients suffering from cardiovascular disease (3, 4, 11, 12). Monocyte subsets characterized by their variable expression of lipopolysaccharide receptor CD14 and Fcγ-III receptor CD16 in peripheral blood have been traditionally divided into classical (CD14++CD16−, M1), intermediate (CD14++CD16+), and non-classical (CD14+CD16+, M2) monocytes (13, 14). Several studies have suggested that increased expression of CD16 either with a greater proportion in intermediate or non-classical monocytes can be used to predict adverse events in patients with heart disease (3, 13). Moreover, in a large study of 951 patients undergoing elective coronary angiography, Rogacev and colleagues were able to demonstrate how elevated values of intermediate monocytes could independently be used to predict long-term adverse cardiovascular outcomes in these patients (4). Similar evidence linking non-classical monocytes to chronic heart failure (HF), particularly HF with normal ejection fraction (EF) have been reported (11, 12, 15). While much of the evidence would suggest that increased CD16 expression is associated with worse outcomes, there remains significant knowledge gaps particularly in patients during heart surgery.

In the present study, we sought to examine and characterize circulating leukocytes in a group of patients undergoing heart surgery for the treatment of ischemic heart disease. More specifically, we hypothesized that circulating leukocytes counts (NLR) and/or more detailed expression of surface markers (CD14 and CD16 expression) can help clinicians in better understanding the burden of inflammation and correlate findings with clinical outcomes.

## Materials and Methods

### Study Population and Objectives

Patients were enrolled prospectively based on admission to the QEII Health Science Centre (Halifax) with ischemic heart disease requiring surgical revascularization or coronary artery bypass grafting (CABG). Informed consent was obtained for all participating patients and all patients received standard care in terms of treatment. Patient enrollment was determined based on participating surgeon and availability of the laboratory team to perform immediate processing of blood and tissue sampling. This also meant that the majority of patients had a history of prior acute coronary syndrome (ACS) and were recovering from their myocardial injury at the time of study. The exclusion criteria included the need for emergent surgery, severe left ventricular dysfunction, and planned concomitant surgery. The present investigation represents findings from a sub-study of a research program called Restitution Enhancement in Arthritis and Chronic Heart disease (REACH), in which the objective was to study mechanisms that would favor resolution of myocardial inflammation in patients with ischemic injury and heart disease. The objectives of the present investigation were to characterize circulating leukocyte and monocyte populations based on CD14 and CD16 expression in patients enrolled in the REACH study. Blood samples were drawn on the day of surgery (day 0), prior to discharge from hospital (day 5), and at the follow-up appointment (day 90). Samples were all processed immediately for flow cytometry and processed for future analysis using standard bio-banking approaches.

### Study Procedures

#### Intraoperative Management

In all patients, cardiac surgery was performed with CPB and anticoagulation was achieved using intravenous heparin given at a dose of 400 IU/kg with a target activated clotting time greater than 450 s. Antifibrinolytic agents were given to all patients and consisted mainly of tranexamic acid. Intermittent cold blood cardioplegia was delivered in an antegrade or retrograde fashion based on surgeon preference. Protamine sulfate was given for reversal of heparin in all patients.

Patients also received routine baseline 12-lead electrocardiograms upon admission to the cardiovascular intensive care unit. Resumption of routine postoperative medications occurred as indicated and included anti-platelet agents within 24 h, statins, and β-blockers.

#### Variable Selection

Preoperative clinical characteristics of interest included age, gender, New York Heart Association (NYHA) functional class, urgency of surgery (urgent if required within 24 h, in-hospital urgent if the patient required hospitalization until the time of surgery, and elective or outpatient), and diabetes. Intraoperative variables included pump time and clamp time. Blood samples were obtained pre-surgery, 5–7 days post-surgery, and >42 days post-surgery. Standard blood analysis, including complete blood counts, NLR, platelet-to-lymphocyte ratio, peak troponin (collected within 24 h of surgery), and monocyte counts were obtained.

#### Blood Processing and Flow Cytometry

In brief, heparinized blood samples (100 μL) were incubated with anti-human antibodies linked to different fluorescent labels, CD45-PerCp-Cy5.5 (BioLegend, San Diego, CA, USA), CD11b-FITC (BioLegend, San Diego, CA, USA), CD14-PE (eBioscience, San Diego, CA, USA), and CD16-APC (eBioscience, San Diego, CA, USA). Labeled whole blood samples were then incubated with 1× FACS Lysing Solution (BD Biosciences, San Jose, CA, USA) in order to lyse red blood cells. Samples were then fixed with 1% paraformaldehyde solution prepared in PBS with 0.1% sodium azide. Isotype controls were used for negative gating to identify the level of non-specific binding. Data were acquired using the FACS Calibur (BD Biosciences, San Jose, CA, USA) then analyzed using FlowJo (FlowJo LLC, Ashland, OR, USA). Monocyte gating was performed by gating on cells with the use of forward and side scatter (FSC and SSC), which was then applied to a CD45 × SSC dot plot. The CD45+ events were gated and applied to a CD11b × SSC dot plot. The CD11b+ events were gated and applied on a CD16 × CD14 dot plot for characterization of distinct monocyte subsets and CD16 mean fluorescence index (MFI).

### Clinical Data Definitions and Outcomes

Clinical data collection and outcomes were recorded using standard case report forms. Follow-up was chosen based on predicted clinical course of patients after myocardial injury that underwent CABG surgery. This means an initial myocardial injury characterized at the time of enrollment followed by clinical improvement and resolution (6 weeks to 3 months). Biological samples were used to study cellular markers of inflammation. The clinical outcomes of interest focused on adverse cardiovascular events (AE). We chose a composite outcome, which included prolonged hospitalization (length of stay >9 days), readmission to hospital after discharge for cardiac reason, persistent HF at follow-up NYHA class III or IV, and inability to wean diuretics for symptoms of congestion (dyspnea, peripheral edema).

### Data Analysis

All data analysis was performed using GraphPad Prism 6 (GraphPad Software Inc., La Jolla, CA, USA). Categorical variables were reported as frequencies and percentages and were analyzed by chi-square.

### Ethics

This study was conducted with the full approval of the institutional (Nova Scotia Health Authority) Research Ethics Board. All personal identifiers were stripped prior to data analysis to ensure patient anonymity and confidentiality.

## Results

### Patient Characteristics

During the study period, a total of 25 patients were screened and approached for consent with 17 patients consenting; yielding a 68% enrollment rate as our study population (Table 1). The majority of patients were males (76.5%), with normal EF (70.6%), requiring surgery while in hospital after a recent myocardial infarction (non-ST elevation myocardial infarction 82.4%), undergoing surgical coronary revascularization or CABG surgery (100%). All patients received the same standard of care.

**Table 1:** **Patient characteristics**.

Parameters		Patients (*n* = 17)
Age		65.9 (44–84)
Female gender		4/17
Diabetes		3/17
Ejection fraction (EF)		60.1 (40–86)
EF > 50%		14/17
Atrial fibrillation		1/17
BMI		28.4 (20–33.3)
	Normal	4/17
	Pre-obese	7/17
	Obese	6/17
Urgency	In-hospital	14/17
	Elective	3/17
Procedure type	Coronary artery bypass grafting (CABG)	16/17
	CABG + aortic valve	1/17
Clinical frailty	CFS class 4/5	7/17
Echocardiogram	LV diast diameter	4.7 (4–5.5)
	LV syst diameter	2.8 (2.2–3.4)
	Left atrium diameter	4 (2.8–4.9)
	E/A ratio	1.2 (0.5–2)

### In-Hospital Findings

**Figure 1:**
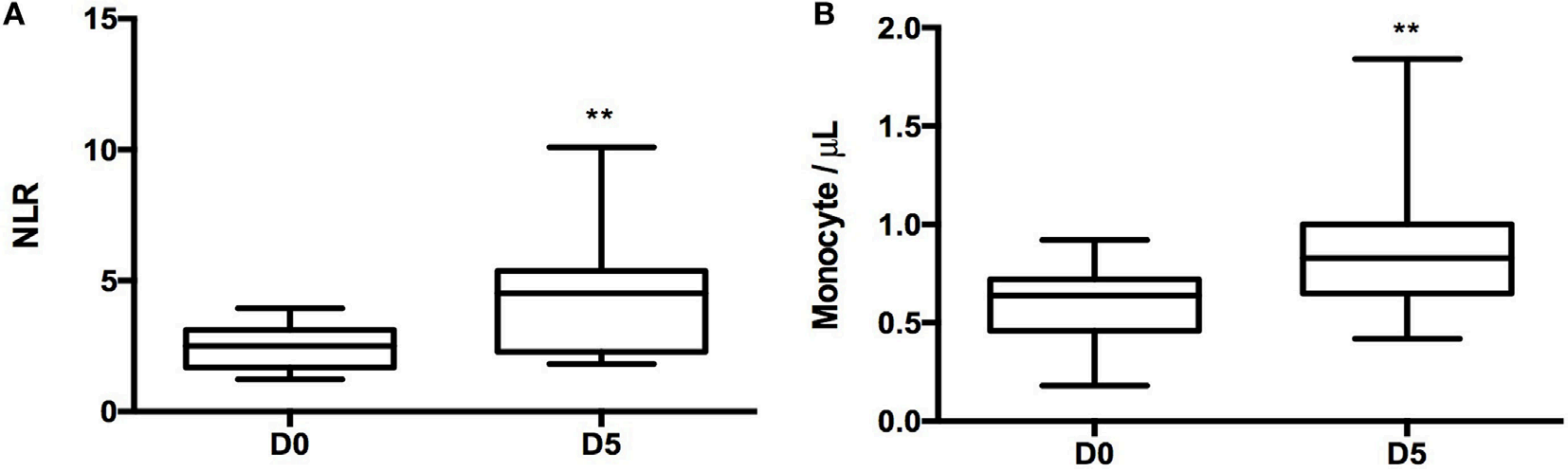
**Neutrophil-to-lymphocyte ratio (NLR) and monocyte change 5 days post-surgery**. **(A)** *T*-test was performed to compare NLR between day 0 and day 5 samples (*p* = 0.0028). **(B)** Monocyte counts per microliter in day 0 and day 5 samples (*p* = 0.0074). For statistical analysis, two-tailed unpaired *t*-test was used; *P < 0.05, **P < 0.01, and ***P < 0.001.

There was no in-hospital mortality and median length of hospitalization was 9 days (IQR). Complete blood counts were obtained from all patients prior to surgery and at a median of 5 days after surgery (Table 2). Leukocyte counts (WBC count) did not change significantly between day 0 and day 5 (Table 2). NLR, the ratio between neutrophils and lymphocytes in circulating blood, has been used by some investigators as a marker of inflammation (1, 5, 9). There was a significant 2.0-fold increase in NLR between baseline and 5 days post-surgery (*p* = 0.0028, Figure 1A). Similarly, there was a 1.7-fold increase in monocyte concentration in blood between baseline and 5 days after surgery (*p* = 0.0074, Figure 1B).

**Table 2:** **Leukocyte counts**.

	Day 0	Day 5	*p*
WBC (×10^9^ cells/L)	6.9 + 0.5	8.4 + 0.6	0.08
Neutrophil (×10^9^ cells/L)	4.3 + 0.3	5.7 + 0.5	0.04*
Lymphocyte (×10^9^ cells/L)	1.9 + 0.2	1.5 + 0.2	0.06
Monocyte (×10^9^ cells/L)	0.6 + 0.05	0.9 + 0.08	0.0074*
Platelet (×10^9^ cells/L)	235.8 + 15.0	228.0 + 23.7	0.78

For statistical analysis, t-test was used; *P < 0.05.

**Figure 2:**
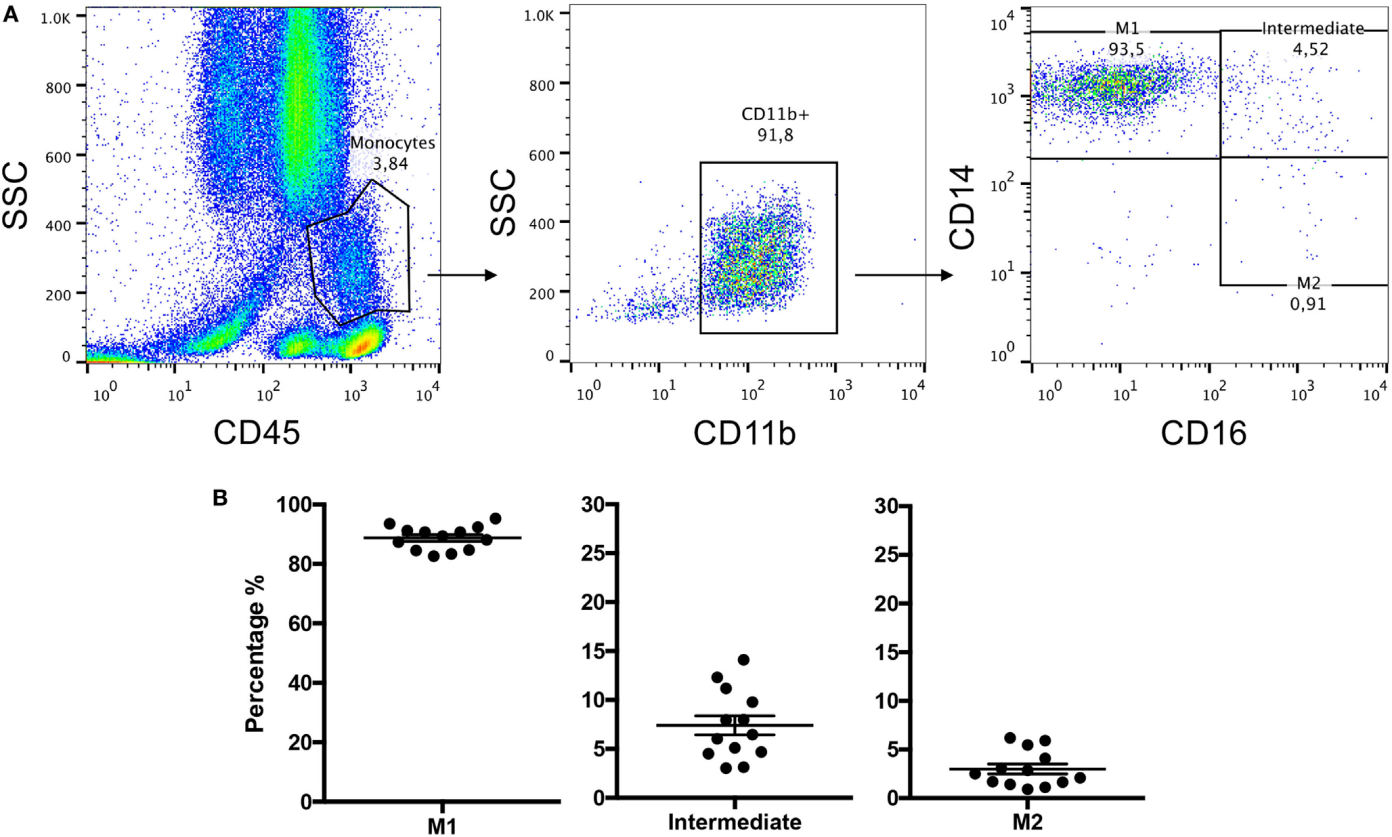
**Whole blood leukocytes isolated from patients’ blood samples were stained with CD11b, CD45, CD14, and CD16 and frequencies of monocyte subsets were measured by flow cytometry**. **(A)** Representative images of flow cytometry gating strategy used to identify monocyte phenotypes from whole leukocyte blood samples. **(B)** Represents subpopulation percentage findings from 13 patients in terms of M1, intermediate, and M2 at day 0 or just prior to surgery.

**Figure 3:**
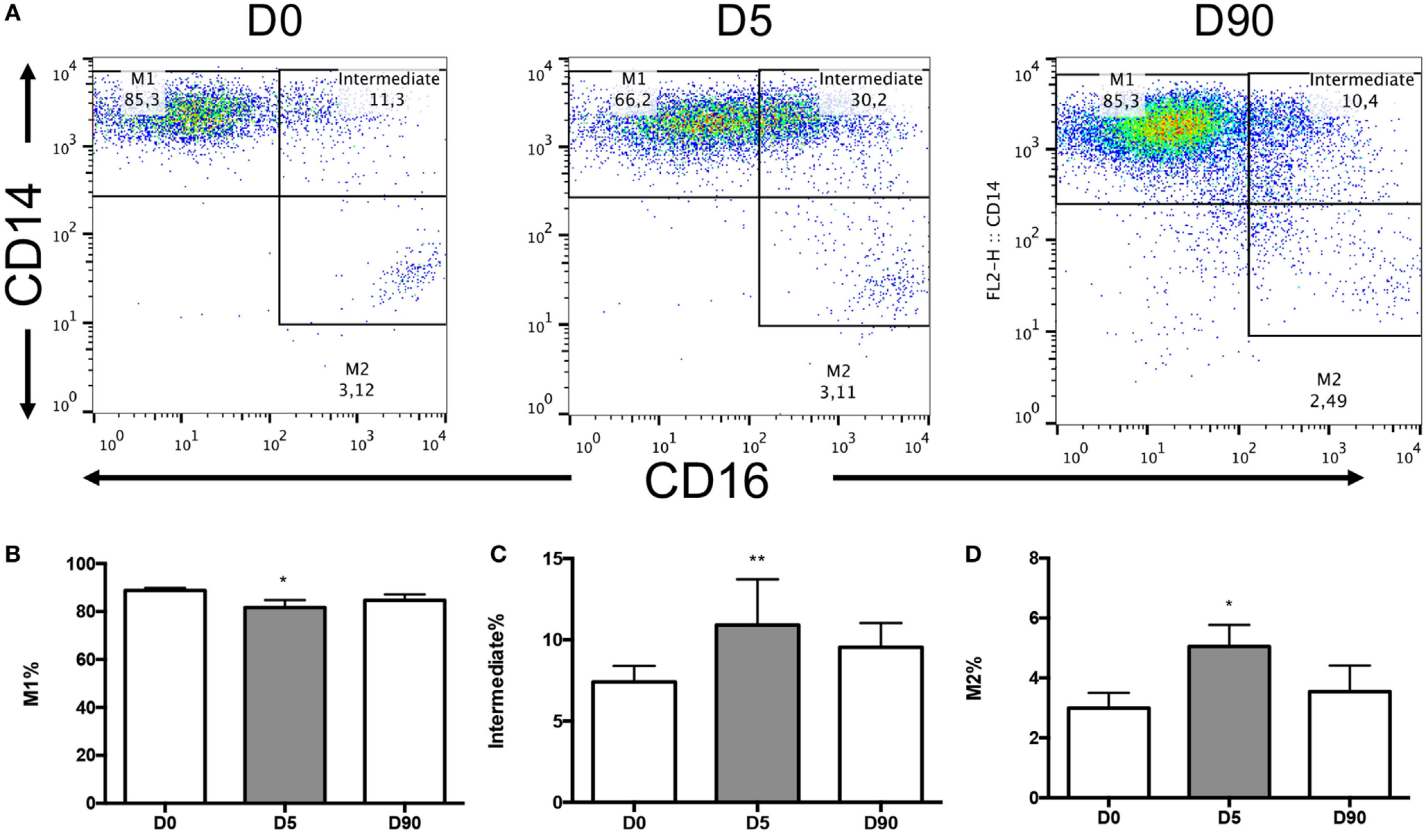
**Monocyte subsets changes over time in circulating blood**. **(A)** The flow cytometry dot plots of an individual patient at day 0 (*n* = 13), day 5 (*n* = 11), and day 90 (*n* = 9) post-surgical intervention. **(B)** Graph demonstrating the change in the M1 monocyte subpopulation over time showing a decrease at day 5 from baseline (*p* = 0.0301). **(C)** Intermediate subpopulation showed a mean 1.47-fold increase at day 5 from baseline (*p* = 0.0025). **(D)** M2 subpopulation was observed to have a 1.69-fold increase at day 5 from baseline (*p* = 0.0257). Day 90 samples were not significantly different from either baseline or day 5 samples. For statistical analysis, two-tailed unpaired *t*-test was used; *P < 0.05, **P < 0.01, and ***P < 0.001.

**Figure 4:**
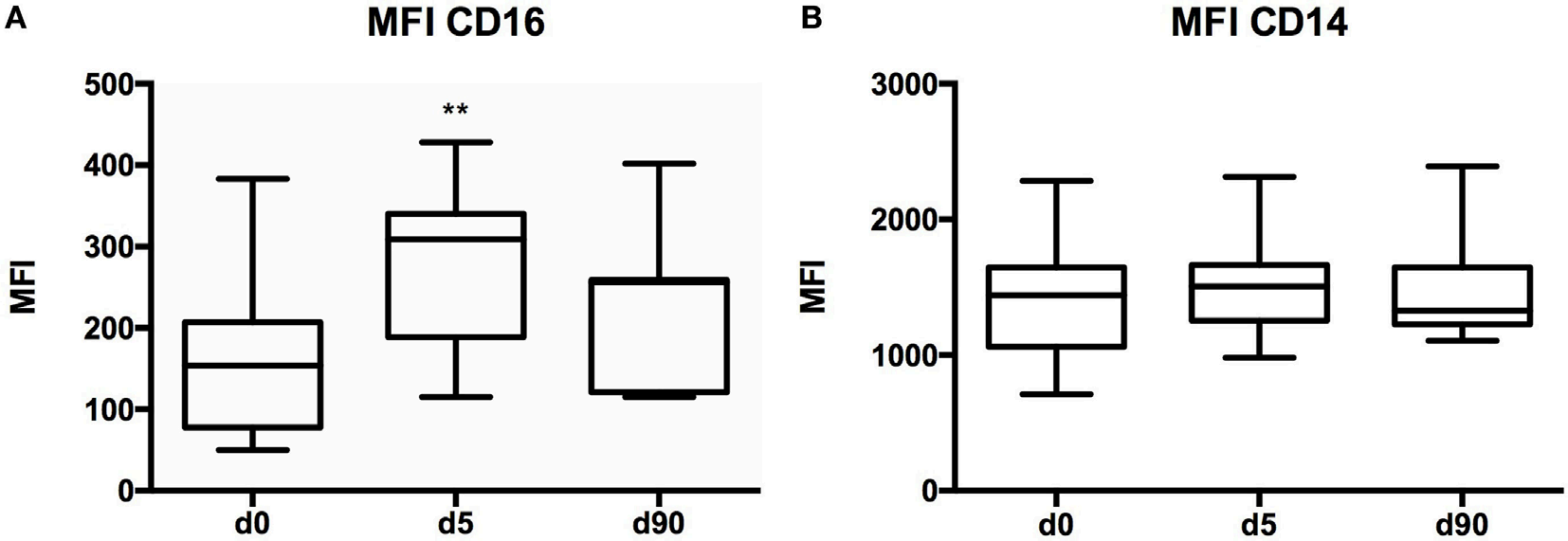
**CD16 and CD14 mean fluorescence index (MFI) values in various time points**. **(A)** Graph showing CD16 MFI differences between the various blood collection time points. There was an increase in CD16 MFI on day 5 when compared to baseline (*p* = 0.0051). **(B)** CD14 MFI was not significantly different between day 0, day 5, and day 90. Day 0 (*n* = 13), day 5 (*n* = 11), and day 90 (*n* = 7) samples. For statistical analysis, two-tailed unpaired *t*-test was used; *P < 0.05, **P < 0.01, and ***P < 0.001.

**Figure 5:**
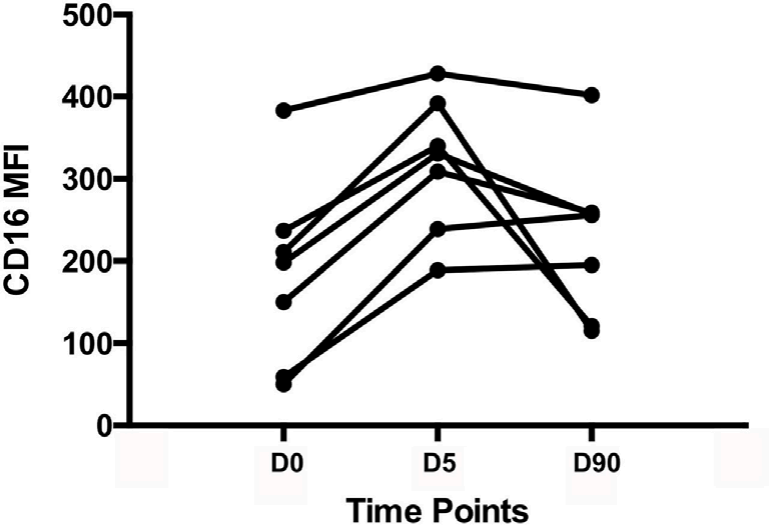
**Individual patient samples**. CD16 mean fluorescence index (MFI) values for individual patients at day 0, day 5, and day 90. It appears that all patients experience an increase in CD16 MFI 5 days post-surgery; however, not all patients return to baseline levels of CD16 MFI at day 90.

A standardized gating strategy was used in order to characterize populations of monocytes (Figure 2). The most abundant monocyte population was the CD14++CD16− (classical, M1) at 88.8 ± 1.1% of all monocytes or also known as classical monocytes. By contrast, CD14++CD16+ (intermediate) population represented 7.4 ± 0.98% of monocytes, and the CD14+CD16+ (non-classical, M2) subset represented 2.99 ± 0.51% of monocytes (Figure 2). Surgery resulted in significant changes in monocyte number and phenotype. A significant expansion of the intermediate and non-classical (M2) populations was observed at the expense of classical (M1) monocytes suggesting a significant shift toward non-classical (M2) monocytes after surgery (Figure 3). Upon analysis of MFI to quantify the overall CD16 expression, surface receptor CD16 MFI was significantly upregulated after surgery (day 5), but returned to baseline levels at long-term follow-up or 90 days after surgery (Figure 4). When we looked at individual patient samples (Figure 5), one can see that increases in CD16 MFI at day 5 are consistent despite different pre-surgery CD16 expression levels. Furthermore, in several patients, CD16 MFI did not seem to return to baseline levels at day 90 and did not appear to correlate with adverse events given the small sample size. CD14 MFI showed no significant differences between pre- and post-surgery suggesting little change in the proportion of classical monocytes (Figure 4).

**Figure 6:**
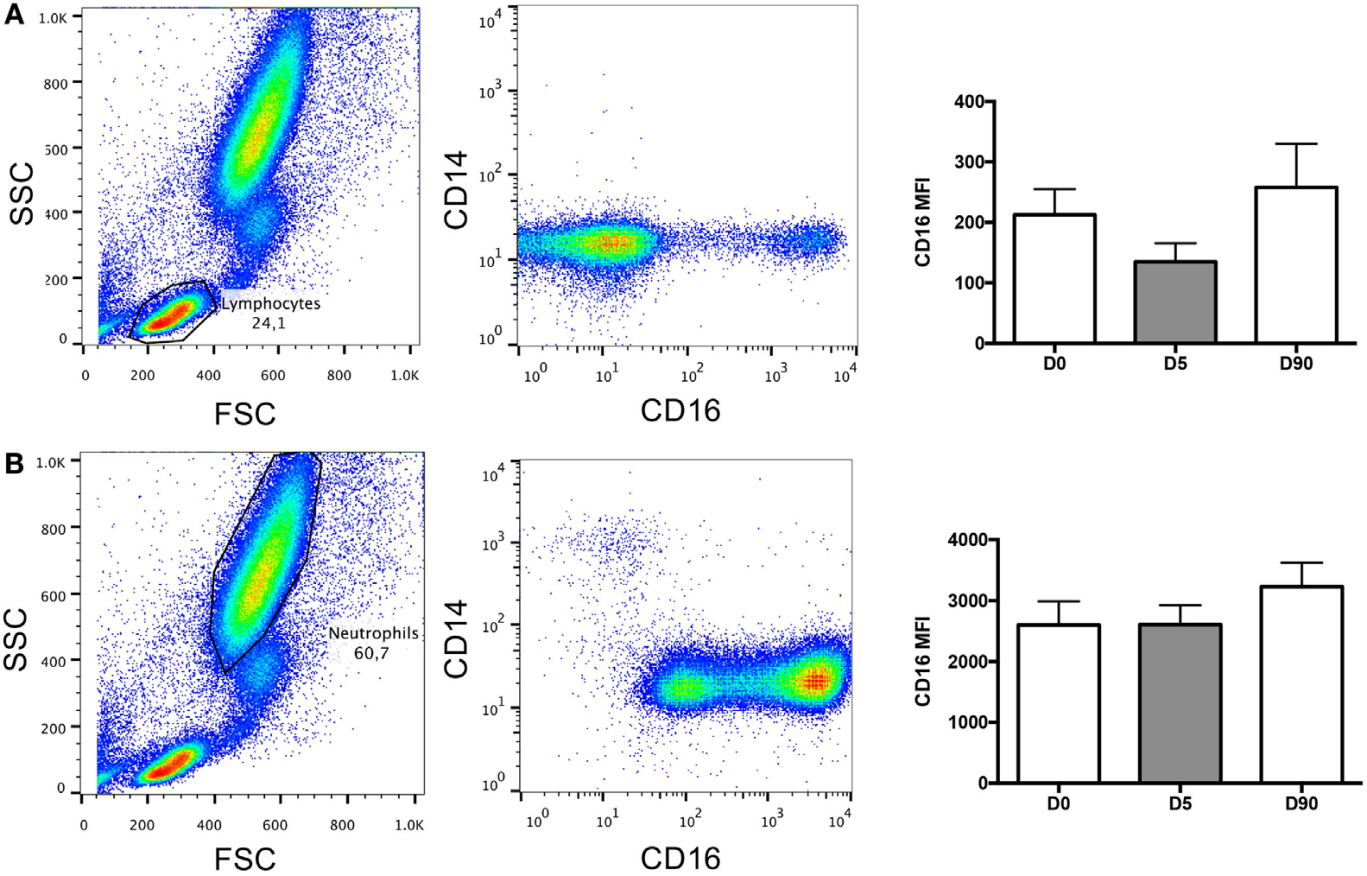
**Lymphocytes and neutrophils contribution to CD16 mean fluorescence index (MFI)**. **(A)** Representative images of flow cytometry dot plots showing the lymphocyte population and its contribution to CD16 expression over time (nanoseconds). **(B)** Representative images of flow cytometry dot plots showing the neutrophil population and its contribution to CD16 expression over time (nanoseconds).

CD16 also known as FcγRIIIA can be expressed on monocytes, macrophages, NK cells, and possibly neutrophils (PMID: 22613847). While our data indicated that CD16 expression was upregulated in monocytes following surgery, we also investigated its expression levels in lymphocytes and neutrophils using flow cytometry. Our findings suggest that CD16 expression was unchanged in both lymphocytes (Figure 6A) and neutrophils (Figure 6B), thus indicating that the elevation in CD16 expression observed post-surgery was specific to circulating monocytes.

**Figure 7:**
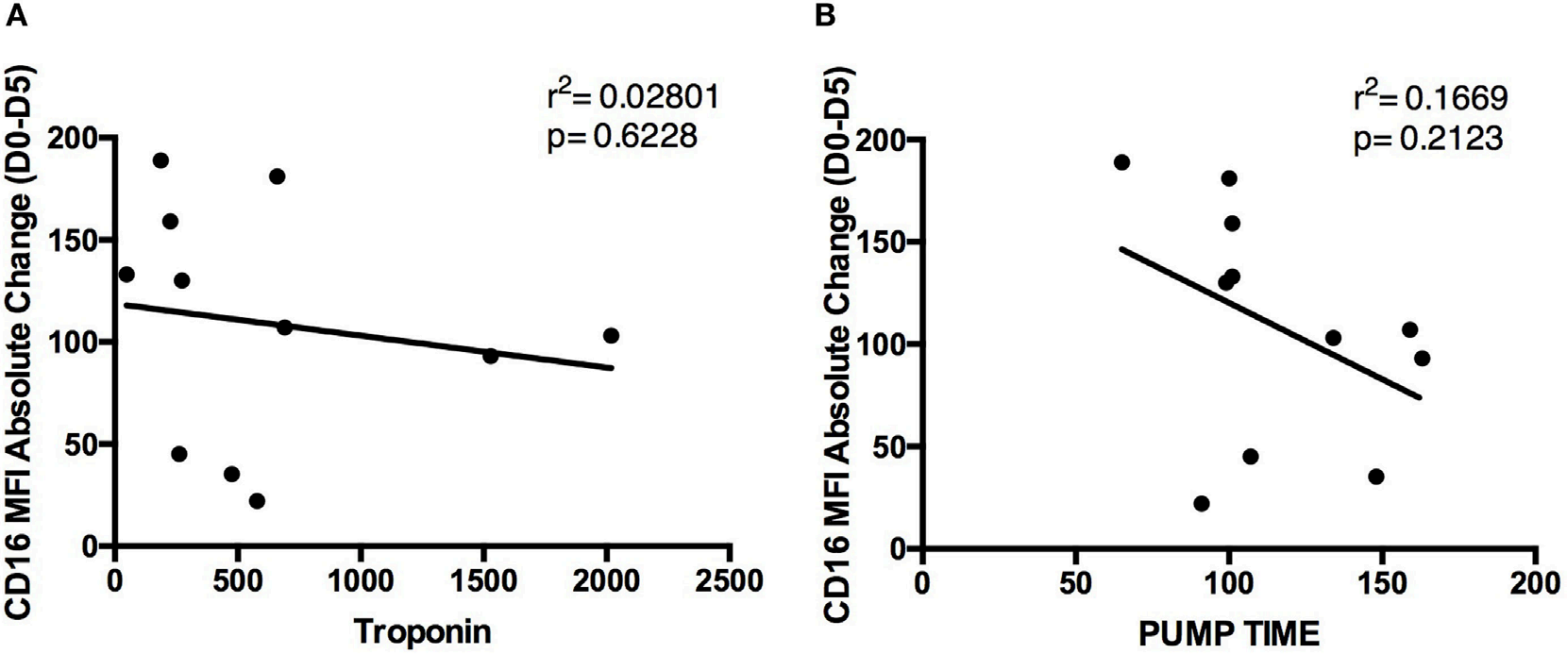
**Relationship of peak troponin and pump time with CD16 mean fluorescence index (MFI) absolute change**. Graphs representing correlations between absolute CD16 MFI change between day 0 and day 5 and **(A)** peak troponin levels and **(B)** pump time. Both parameters were not significant with CD16 MFI (*n* = 11).

To better understand the mechanism by which CD16 expression is influenced by cardiac surgery using CPB, examined the absolute change in MFI pre- and post-surgery. CD16 expression correlated with two well-described variables known to influence inflammation after CPB: myocardial injury (troponin level post-surgery, cTnT nanograms/milliliter) and duration of CPB (pump time) (16). Blood samples for cTnT analysis were obtained for all patients within the first 24 h post-surgery. The peak cTnT levels (highest cTnT value in patients with multiple samples) were found to be 544.1 ± 125.2 ng/mL. Correlation analyses indicated that there was no significant relationship between peak troponin and change in CD16 MFI (Figure 7A) or pump time (minutes) and CD16 MFI values (Figure 7B).

**Figure 8:**
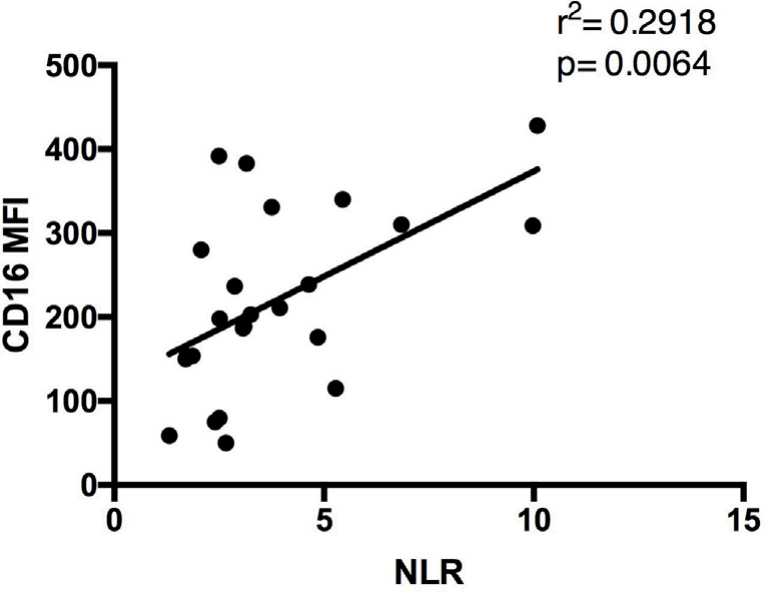
**Relationship between CD16 mean fluorescence index (MFI) and neutrophil-to-lymphocyte ratio (NLR) values**. Graph showing a positive correlation between CD16 MFI and NLR in patients’ blood samples. Sample size included both day 0 and day 5 findings to show the universal relationship between the parameters tested (*n* = 24).

In the present manuscript, we evaluated both NLR and CD16 expression as cellular markers reflecting inflammatory changes that occur in patients undergoing heart surgery. The correlation between NLR and CD16 MFI was assessed (Figure 8). By plotting this relationship, we were able to determine a positive correlation between NLR and CD16 MFI (*r*^2^ = 0.2918, *p* = 0.006), suggesting that higher NLR were associated with increased numbers of non-classical (M2) monocytes in circulation.

### Patient Outcomes and Markers of Inflammation

**Figure 9:**
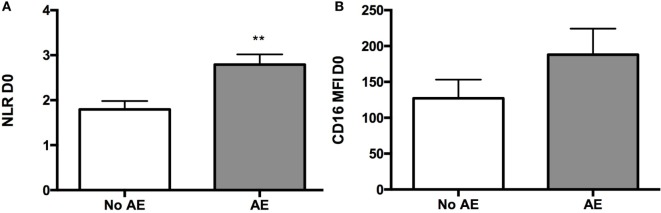
**Differences between patients who developed adverse events post-surgery and patients who did not develop in terms of pre-op blood findings**. **(A)** Graph showing a higher mean NLR value in pre-op samples of patients who developed AE when compared to pre-op samples of patients who did not develop AE post-surgery (*p* = 0.0065). **(B)** Graph representing CD16 mean fluorescence index (MFI) mean values at baseline in the AE and no AE patient groups (no significant difference was observed). For statistical analysis, two-tailed unpaired *t*-test was used; *P < 0.05, **P < 0.01, and ***P < 0.001.

All patients were followed after discharge from hospital for a minimum of 90 days after surgery. Follow-up was complete in 100% of patients with no in-hospital or medium term mortality. Adverse cardiovascular outcome (AE) was defined as any one of the following: prolonged length of hospitalization (LOS) greater than 9 days, readmission to hospital for any cardiac reason, persistent HF at follow-up NYHA class III or IV, and inability to wean diuretics for symptoms of congestion (dyspnea, peripheral edema). A total of 59% (10/17) of patients suffered AE with the majority (47%) of patients hospitalized for longer than 9 days and 12% of patients being readmitted after discharge for cardiac reasons. One patient was readmitted with congestive HF (within 1 week of discharge) and one with an ACS (2 months after discharge). Patient characteristics were not significantly different between patients with AE (*n* = 10) and those without AE (*n* = 7) (Table 3). The mean length of stay was 18.9 days in AE patients compared to 6.9 days in those without AE (*p* = 0.041). Additionally, our unadjusted findings suggest that patients who develop AE have a significantly higher NLR value before surgery, when compared to patients who did not develop AE (*p* = 0.007), however, this failed to reach significance for CD16 MFI (*p* = 0.25) (Figure 9). Of note, patients in the AE group were more likely to develop delirium as a complication in hospital (0 versus 50%; *p* = 0.044) and have a significantly higher peak troponin indicating myocardial injury (215.9 + 44.9 versus 773.8 + 179.5 ng/mL; *p* = 0.023).

**Table 3:** **Follow-up patient grouping by development of adverse events**.

Parameters		No AE (*n* = 7)	With AE (*n* = 10)	*p*-Value
Age		65.4 ± 3.7	66.2 ± 4.2	0.89
Female gender		2/7	2/10	1.0
Diabetes		0/7	3/10	0.23
Ejection fraction		63.3 ± 3.7	57.9 ± 4.6	0.41
Atrial fibrillation (pre- and post-op)		3/7	3/10	0.64
BMI		30.4 ± 0.94	27.0 ± 1.5	0.11
	Normal	0/7	4/10	
	Pre-obese	3/7	3/10	
	Obese I/II/III	4/7	3/10	
Urgency	In-hospital	5/7	9/10	0.54
	Elective	2/7	1/10	
Clinical frailty	4/5	1/7	6/10	0.13
Echocardiogram	LV diast	4.7 ± 0.20	4.66 ± 0.15	0.80
	LV syst	2.85 ± 0.05	2.74 ± 0.21	0.79
	Left atrium	4.03 ± 0.44	3.94 ± 0.23	0.83
	E/A	1.14 ± 0.33	1.16 ± 0.19	0.94
Cardiopulmonary bypass	Pump time	97.4 ± 6.1	121.8 ± 10.1	0.085
Delirium		0/7	5/10	0.044*
CHF		0/7	4/10	0.10
LOS	Average	6.9 ± 0.5	18.9 ± 4.5	0.041*
	PLOS > 9 days	0/7	8/10	0.0023*
Peak trop		215.9 ± 44.9	773.8 ± 179.5	0.023*

For statistical analysis, chi-squared test was used; *P < 0.05.

## Discussion

**Figure 10:**
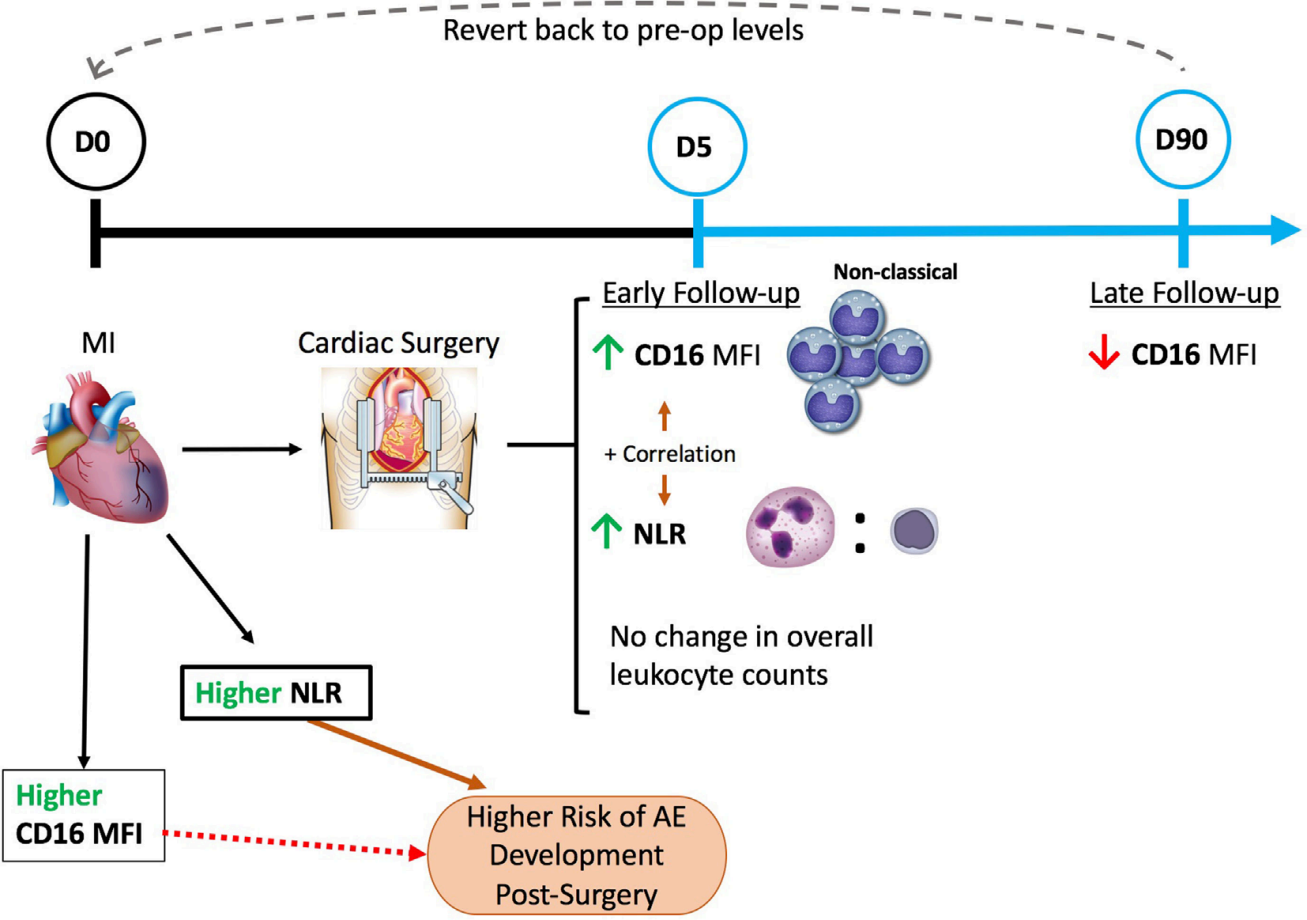
**A schematic of our major findings and understanding of patient circulating leukocyte relation to cardiac surgery**. Cardiac surgery with cardiopulmonary bypass increased the baseline levels of CD16 expression on circulating monocytes (non-classical) and neutrophil-to-lymphocyte ratio (NLR) values seen 5 days post-surgery with no change in overall leukocyte counts. Moreover, a positive correlation between CD16 mean fluorescence index (MFI) and NLR was evident. At a later follow-up time-point (day 90), a downregulation of CD16 expression was observed that seems to revert to pre-op levels of expression. Pre-op NLR was shown to be significantly higher in patients that later developed AE than patients who did not, therefore, putting patients with higher NLR values pre-op at a higher risk of developing AE. Conversely, CD16 values were not significantly different between the two groups, however, showed a trend similar to NLR.

It is increasingly recognized that inflammation plays a crucial role and may be responsible for disease progression in conditions like cardiovascular disease (17, 18). Similarly, inflammation has proven to be an important component of the normal host response to injury and necessary for healing. This seemingly conflicting role of inflammation highlights how clinically relevant efforts in this area remain (18). However, to date our ability to qualify or quantify inflammation using simple biomarkers remains poorly defined. In the present study, we used peripheral blood to characterize circulating leukocytes namely monocytes and NLR. NLR was used as a marker of cellular inflammatory changes that occur in a group of patients undergoing heart surgery for the treatment of ischemic heart disease with the majority of patients having suffered an ACS within 21 days of surgery. Specifically, we demonstrated a significant increase in NLR after surgery without a significant change in overall leukocyte counts. We demonstrated significant increases in overall neutrophil and monocyte counts at the expense of lymphocyte counts. Our observation is not unique and has been previously described (10, 19). What is novel about our observations is that we also correlated our findings with monocyte subsets based on CD16 expression. We demonstrated that NLR is significantly correlated with CD16 expression on circulating monocytes (Figure 10).

While not fully understood, monocyte-CD16 expression has been suggested to be a marker of maturity, sharing many features with tissue macrophages that have been described as non-classical M2 macrophages (3). It may therefore suggest that this persistence of non-classical M2 cells follows a heightened (NLR) preoperative state of inflammation that is unresolved postoperatively and thus contributing to AE. Our understanding of circulating monocytes and their phenotype has potentially important implications, as circulating monocytes are precursor cells to tissue macrophages. In the context of heart disease, increasing efforts have been made to better understand the balance between seemingly opposite pro-inflammatory M1 macrophages and non-classical macrophages M2 (20). M2 macrophages exhibit functional characteristics that can exert opposing effects to classical M1 macrophages, such as an anti-inflammatory (IL-10) and/or pro-fibrotic cytokine production (TGF-β) normally associated with healing or fibrosis (21, 22). A proposed beneficial role for the M2 subset (non-classical) is likely an oversimplification, given the heterogeneity of this population (12, 14, 20). One should note that some groups have suggested that larger proportions of classical CD16 monocytes would be more predictive, but this was not the case in our study as the expression of CD14 did not change after surgery from baseline levels in our patient samples, indicating that classical (M1) monocytes were not predictive (23). The full significance of this observation remains to be determined; however, it could be that persistence of CD16 is indicative of a failure to resolve inflammation by M2 macrophages (providing negative feedback regulation) in the normal course of healing.

To date the use of CD16 expression as a biomarker to identify patients at risk of cardiovascular complications has yet to be widely applied and remains limited to case series (3, 4, 11). We argue that an important reason for flow cytometric analyses of blood have not been routinely clinically used is at least partly related to the fact that such assays are time consuming, labor intensive, and as such very expensive. To the best of our knowledge, we are the first to demonstrate a significant correlation between CD16 expression and clinical NLR values. Determining NLR, in contrast to CD16 MFI, can be obtained simply from a complete blood count. Complete blood counts are readily available in all patients undergoing surgery and are therefore convenient. Previous studies have demonstrated that NLR can be a useful biomarker for predicting adverse cardiovascular events after cardiac surgery or acute coronary events (1, 2, 5, 9). In our small study, we report that NLR at the time of surgery was predictive of an increased likelihood in developing AE after surgery supporting its use as a potentially useful biomarker to predict outcomes. One should note that in contrast to NLR, CD16 MFI was not predictive of AE despite showing a trend.

Despite having identified a significant correlation between NLR and CD16 MFI, we note that this is a small study and as such adjusting to potential differences between patients was not possible. As such, it may be that our patients were similar in terms of characteristics but our sample size was too small to identify significant differences at this stage. Previously, cardiac surgery patients that developed AE were sixfold more likely to be frail at the time of surgery (24). Clinical frailty at the time of cardiac surgery has been shown by our group to be a major risk factor for AE also (25). It is possible that NLR findings in this study not only reflect patient’s inflammatory state at the time of surgery, but could also reflect an association between inflammation and frailty.

An important observation from this study is the fact that prolonged hospitalization was the most common cause of AE (80%) and driven mainly by significant postoperative delirium (*p* = 0.044). Delirium is a complex clinical condition that manifests clinically as an acute confused state characterized by fluctuating mental status, inattention, and either disorganized thinking or altered level of consciousness (26). Relevant to the present manuscript are reports linking delirium with inflammation, but the mechanisms for this relationship require further study (27).

In the present manuscript, we focused our attention on the phenotype of circulating leukocytes largely based on increasing evidence linking them to cardiovascular outcomes (1–5, 11, 12, 24). To the best of our knowledge, we are the first to describe changes in CD14 and CD16 expression pre- and post-surgery, as well at later follow-up. Our findings suggest that circulating monocytes undergo significant phenotypic changes toward M2 phenotype early after surgery at the expense of M1 phenotype. The mechanism for this change has yet to be determined and is beyond the scope of the present manuscript, but does highlight the need to better understand the functional significance of the observation beyond the association with AE as we have suggested. Others have described significant changes in cell surface expression of other markers of activation (HLA-DR, CD163, TM) of various subsets of immune cells like lymphocytes, B cells, and monocytes associated with CPB supporting our findings, but like others we do not fully understand its implications (7, 28, 29). It is noteworthy that the changes we observed early after cardiac surgery did resolve over time suggesting that some of these changes may not only be related to the surgery, but may also be important in healing and/or involved in regulating either the initial inflammatory response or its resolution.

While our findings are novel they are significantly limited by the study’s small size, patient heterogeneity, biological complexity of ACS patients undergoing cardiac surgery, and our inability to compare to a non-surgical control population. Despite these limitations, we provided evidence supporting the routine use of NLR as a surrogate for changes in monocyte phenotype associated with cardiac surgery. Given previous work linking monocyte phenotype and NLR to patient outcomes, we believe the present manuscript offers important supportive evidence for these biomarkers warranting further mechanistic studies. Future studies will attempt to better characterize the function of isolated leukocytes/monocytes from patients (high versus low NLR) using tools available to our group, which includes coculture systems to test effects of inflammation on fibroblasts and fibrosis.

## Author Contributions

This study “REACH-OPOS” was designed, directed, and coordinated by KG, SL, JM, AH, KB, PK, TP, and J-FL. KG performed and analyzed cell isolations and FACS staining. TM and CW performed and analyzed the immunohistochemistry and Sirius Red experiments. JM, KB, PK, and TP aided with further interpretation of the results. The manuscript was written by KG and J-FL and commented on by all authors.

## Conflict of Interest Statement

The authors declare that the research was conducted in the absence of any commercial or financial relationships that could be construed as a potential conflict of interest.

## Abbreviations

AE, adverse cardiovascular event; CABG, coronary artery bypass grafting; CPB, cardiopulmonary bypass; CIHR, Canadian Institute of Health Research; COPD, chronic obstructive pulmonary disease; Cr, serum creatinine; CRP, C-reactive protein; CVICU, Cardiovascular Intensive Care Unit; EF, ejection fraction; FSC, forward scatter; HF, heart failure; IL-1β, interleukin one beta; LA, left atrium; MFI, mean fluorescence index; NLR, neutrophil-to-lymphocyte ratio; NSTEMI, non-ST elevation myocardial infarction; NYHA, New York Heart Association; PLR, platelet-to-lymphocyte ratio; QEII HSC, Queen Elizabeth II Health Sciences Centre; REACH, Restitution Enhancement in Arthritis and Chronic Heart disease; SSC, side scatter; STEMI, ST elevation myocardial infarction; TNFα, tumor necrosis factor alpha.

## References

[1] Tan TP Arekapudi A Metha J Prasad A Venkatraghavan L. Neutrophil-lymphocyte ratio as predictor of mortality and morbidity in cardiovascular surgery: a systematic review. ANZ J Surg (2015) 85(6):414–9. 10.1111/ans.13036 25781147

[2] Kim WH Park JY Ok SH Shin IW Sohn JT. Association between the neutrophil/lymphocyte ratio and acute kidney injury after cardiovascular surgery: a retrospective observational study. Medicine (Baltimore) (2015) 94(43):e1867. 10.1097/MD.0000000000001867 26512598 PMC4985412

[3] Idzkowska E Eljaszewicz A Miklasz P Musial WJ Tycinska AM Moniuszko M. The role of different monocyte subsets in the pathogenesis of atherosclerosis and acute coronary syndromes. Scand J Immunol (2015) 82(3):163–73. 10.1111/sji.12314 25997925

[4] Rogacev KS Cremers B Zawada AM Seiler S Binder N Ege P CD14++CD16+ monocytes independently predict cardiovascular events: a cohort study of 951 patients referred for elective coronary angiography. J Am Coll Cardiol (2012) 60(16):1512–20. 10.1016/j.jacc.2012.07.019 22999728

[5] Gibson PH Croal BL Cuthbertson BH Small GR Ifezulike AI Gibson G Preoperative neutrophil-lymphocyte ratio and outcome from coronary artery bypass grafting. Am Heart J (2007) 154(5):995–1002. 10.1016/j.ahj.2007.06.043 17967611

[6] Sun Y Yi D Wang Y Zheng R Sun G Wang J Age-dependent mobilization of circulating endothelial progenitor cells in infants and young children undergoing cardiac surgery with cardiopulmonary bypass. Cytokine (2009) 47(3):206–13. 10.1016/j.cyto.2009.06.009 19632131

[7] Tsai CS Tsai YT Lin CY Lin TC Huang GS Hong GJ Expression of thrombomodulin on monocytes is associated with early outcomes in patients with coronary artery bypass graft surgery. Shock (2010) 34(1):31–9. 10.1097/SHK.0b013e3181d494c4 20090566

[8] Landis RC Brown JR Fitzgerald D Likosky DS Shore-Lesserson L Baker RA Attenuating the systemic inflammatory response to adult cardiopulmonary bypass: a critical review of the evidence base. J Extra Corpor Technol (2014) 46(3):197–211. 26357785 PMC4566828

[9] Guasti L Dentali F Castiglioni L Maroni L Marino F Squizzato A Neutrophils and clinical outcomes in patients with acute coronary syndromes and/or cardiac revascularisation. A systematic review on more than 34,000 subjects. Thromb Haemost (2011) 106(4):591–9. 10.1160/TH11-02-0096 21866299

[10] Gennari R Dominioni L Imperatori A Bianchi V Maroni P Dionigi R. Alterations in lymphocyte subsets as prognosticators of postoperative infections. Eur J Surg (1995) 161(7):493–9. 7488663

[11] Wrigley BJ Shantsila E Tapp LD Lip GY. CD14++CD16+ monocytes in patients with acute ischaemic heart failure. Eur J Clin Invest (2013) 43(2):121–30. 10.1111/eci.12023 23240665

[12] Glezeva N Voon V Watson C Horgan S McDonald K Ledwidge M Exaggerated inflammation and monocytosis associate with diastolic dysfunction in heart failure with preserved ejection fraction: evidence of M2 macrophage activation in disease pathogenesis. J Card Fail (2015) 21(2):167–77. 10.1016/j.cardfail.2014.11.004 25459685

[13] Tsujioka H Imanishi T Ikejima H Kuroi A Takarada S Tanimoto T Impact of heterogeneity of human peripheral blood monocyte subsets on myocardial salvage in patients with primary acute myocardial infarction. J Am Coll Cardiol (2009) 54(2):130–8. 10.1016/j.jacc.2009.04.021 19573729

[14] Nahrendorf M Swirski FK. Monocyte and macrophage heterogeneity in the heart. Circ Res (2013) 112(12):1624–33. 10.1161/CIRCRESAHA.113.300890 23743228 PMC3753681

[15] Barisione C Garibaldi S Ghigliotti G Fabbi P Altieri P Casale MC CD14CD16 monocyte subset levels in heart failure patients. Dis Markers (2010) 28(2):115–24. 10.3233/DMA-2010-0691 20364047 PMC3833331

[16] Mokhtar AT Begum J Buth KJ Legare JF Cardiac troponin T is an important predictor of mortality after cardiac surgery. J Crit Care (2016) 38:41–6. 10.1016/j.jcrc.2016.10.011 27837691

[17] Dick SA Epelman S Chronic heart failure and inflammation: what do we really know? Circ Res (2016) 119(1):159–76. 10.1161/CIRCRESAHA.116.308030 27340274

[18] Ruparelia N Chai JT Fisher EA Choudhury RP Inflammatory processes in cardiovascular disease: a route to targeted therapies. Nat Rev Cardiol (2016) 14:133–44. 10.1038/nrcardio.2016.185 27905474 PMC5525550

[19] Tamhane UU Aneja S Montgomery D Rogers EK Eagle KA Gurm HS. Association between admission neutrophil to lymphocyte ratio and outcomes in patients with acute coronary syndrome. Am J Cardiol (2008) 102(6):653–7. 10.1016/j.amjcard.2008.05.006 18773982

[20] Falkenham A de Antueno R Rosin N Betsch D Lee TD Duncan R Nonclassical resident macrophages are important determinants in the development of myocardial fibrosis. Am J Pathol (2015) 185(4):927–42. 10.1016/j.ajpath.2014.11.027 25794704

[21] Nahrendorf M Swirski FK Aikawa E Stangenberg L Wurdinger T Figueiredo JL The healing myocardium sequentially mobilizes two monocyte subsets with divergent and complementary functions. J Exp Med (2007) 204(12):3037–47. 10.1084/jem.20070885 18025128 PMC2118517

[22] Pilling D Fan T Huang D Kaul B Gomer RH. Identification of markers that distinguish monocyte-derived fibrocytes from monocytes, macrophages, and fibroblasts. PLoS One (2009) 4(10):e7475. 10.1371/journal.pone.0007475 19834619 PMC2759556

[23] Berg KE Ljungcrantz I Andersson L Bryngelsson C Hedblad B Fredrikson GN Elevated CD14++CD16− monocytes predict cardiovascular events. Circ Cardiovasc Genet (2012) 5(1):122–31. 10.1161/CIRCGENETICS.111.960385 22238190

[24] Dent E Kowal P Hoogendijk EO. Frailty measurement in research and clinical practice: a review. Eur J Intern Med (2016) 31:3–10. 10.1016/j.ejim.2016.03.007 27039014

[25] Bagnall NM Faiz O Darzi A Athanasiou T. What is the utility of preoperative frailty assessment for risk stratification in cardiac surgery? Interact Cardiovasc Thorac Surg (2013) 17(2):398–402. 10.1093/icvts/ivt197 23667068 PMC3715194

[26] Jung P Pereira MA Hiebert B Song X Rockwood K Tangri N The impact of frailty on postoperative delirium in cardiac surgery patients. J Thorac Cardiovasc Surg (2015) 149(3):869–75.e1–2. 10.1016/j.jtcvs.2014.10.118 25486976

[27] Vasunilashorn SM Ngo L Inouye SK Libermann TA Jones RN Alsop DC Cytokines and postoperative delirium in older patients undergoing major elective surgery. J Gerontol A Biol Sci Med Sci (2015) 70(10):1289–95. 10.1093/gerona/glv083 26215633 PMC4817082

[28] Holmannova D Kolackova M Kunes P Krejsek J Mandak J Andrys C. Impact of cardiac surgery on the expression of CD40, CD80, CD86 and HLA-DR on B cells and monocytes. Perfusion (2016) 31(5):391–400. 10.1177/0267659115612905 26503949

[29] Kolackova M Kudlova M Kunes P Lonsky V Mandak J Andrys C Early expression of FcgammaRI (CD64) on monocytes of cardiac surgical patients and higher density of monocyte anti-inflammatory scavenger CD163 receptor in “on-pump” patients. Mediators Inflamm (2008) 2008:235461 10.1155/2008/235461 18320015 PMC2248246

